# Bone defect filling with a novel rattan-wood based not-sintered hydroxyapatite and beta-tricalcium phosphate material (b.Bone™) after tricortical bone graft harvesting – A consecutive clinical case series of 9 patients

**DOI:** 10.1016/j.tcr.2023.100805

**Published:** 2023-02-18

**Authors:** Volker Alt, Nike Walter, Markus Rupp, Thierry Begué, Michael Plecko

**Affiliations:** a Department of Trauma Surgery, University Hospital Regensburg, Germany; b Department of Orthopedics and Trauma Surgery, Antoine Beclere Hospital, University Paris-Saclay, France; c AUVA – Trauma Center Styria (UKH) Hospital Graz, Austria

**Keywords:** Biomaterial, Bone defect, Hydroxyapatite, Iliac crest, beta-tricalcium phosphate

## Abstract

Harvesting of tricortical bone graft from the iliac crest is an integral part of bone defect reconstruction in orthopaedic surgery. There are several options for filling the iliac crest defect area to avoid hematoma, pain, hernias and cosmetic issues, including different gelatin-based and other alternative biomaterials. Recently, a novel rattan-wood based not-sintered hydroxyapatite and beta-tricalcium phosphate material (b.Bone™, GreenBone ORTHO S.p.A Faenza, Italy) was shown to promote bone healing in an experimental setting. The goal of the current work is to report clinical and radiographical outcomes of a consecutive case series of 9 patients with defect filling at the iliac crest with this novel scaffold biomaterial after tricortical bone graft harvesting with a minimum follow-up of 6 months. All 9 patients (8 male, 1 female) with an average age of 42.7 years (range: 18–76 years) had tricortical bone graft harvesting from the iliac crest for different reconstructive procedures at the extremities and received blocks of the biomaterial with an average size of 26.3 × 16.8 × 10 mm (length, height, width; range: 15 × 15 × 10 to 40 × 20 × 10 mm). Intraoperative handling of the biomaterial was easy and the blocks could be customized to the individual size of the defect with standard surgical instruments and were press-fitted into the defect. All 9 patients showed uneventful wound healing at the iliac crest and 7 patients reported no pain (VAS: 0) and two patients only mild pain (VAS:1 and VAS:3) after an average follow-up of 9.8 months (range: 6–16 months). There was no post-operative hematoma, surgical revision or other implant-related complications at the iliac crest. In all patients, good radiographical integration without dislocation of the implant and good bony integration was observed. The use of this novel biomaterial for iliac crest defect filling was associated with good clinical and radiographical outcomes after an average follow-up of 9.8 months.

## Introduction

Bone grafting is essential in reconstructive orthopaedic surgery with 54,784 annual procedures in Germany and more than half a million in the United States [1], [2]. The iliac crest is the most common donor site, whereby reconstruction of the iliac crest defects after harvesting reduces complications such as hematoma, pain, and hernias [3]. There are several options for defect filling of the iliac crest and numerous biomaterials have been developed in the last twenty years, particularly based on hydroxyapatite (HA) and other calcium phosphates (CaPs) [4]. Recently, also innovative processes were introduced using rattan wood as a template for a biomorphic transformation into 3D bone-like hierarchical structure [5], [6], [7], [8]. Consequently, such novel rattan-wood based not-sintered hydroxyapatite and beta-tricalcium phosphate material has been launched to the market (b.Bone™, GreenBone ORTHO S.p.A Faenza, Italy). Experimental studies have already confirmed a high level of structural mimicry by the scaffold and showed strongly enhanced osteogenic potential in vitro [9], [10], [11]. However, only one case with the implantation of this scaffold into the iliac crest [12] has been published, but clinical data on the safety and performance of the rattan-wood-derived scaffold within larger series have not been reported so far.

Thus, here, we describe the clinical and radiographical outcomes of a consecutive case series of 9 patients with defect filling at the iliac crest with the GreenBone scaffold biomaterial after tricortical bone graft harvesting.

## Clinical case series

In total, n = 9 patients (8 male, 1 female) with an average age of 42.7 years (range: 18–76 years) underwent tricortical bone graft harvesting from the iliac crest for different reconstructive procedures at the extremities, such as shoulder stabilization, non-union of long bones etc. with the harvested tricortical graft at three different centres. The first centre included five, the second centre included three and the third centre included one patient.

**Fig. 1:**
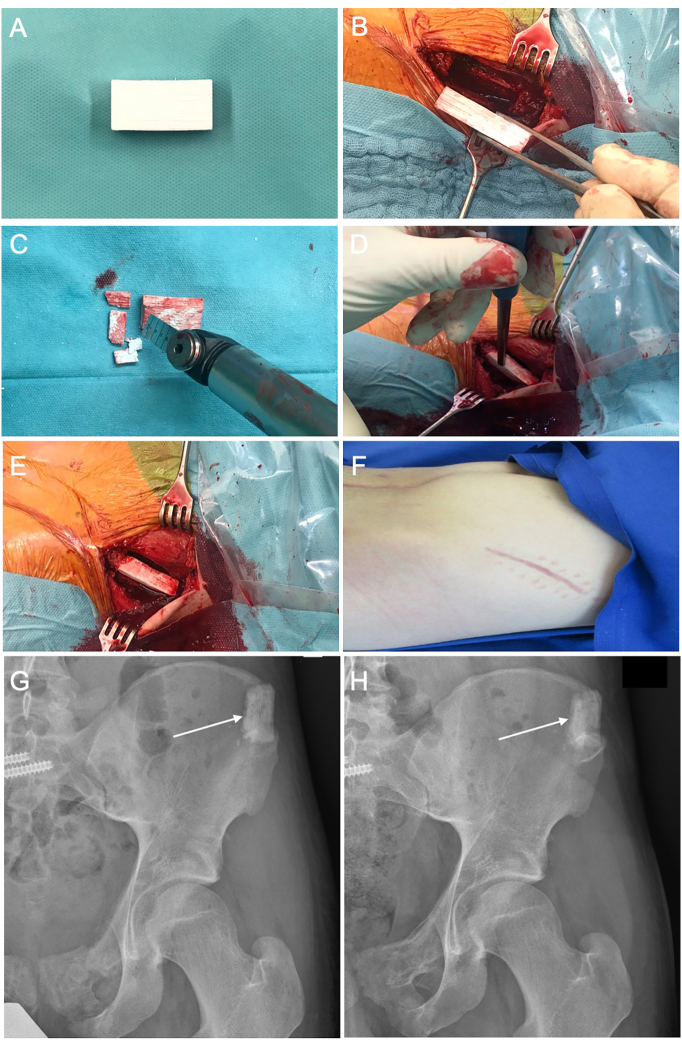
Block shaped scaffold of rattan-wood based not-sintered hydroxyapatite and beta-tricalcium phosphate material (b.Bone™, GreenBone ORTHO S.p.A Faenza, Italy) with a size of 40x30x10 mm (A). Defect at the left iliac crest after tricortical bone graft harvesting with a defect size of 30 × 20 × 10 mm. Customizing of the biomaterial with oscillating saw for press-fit implantation by shortening of the block by 9 mm (C) and press-fit insertion of the scaffold into the defect with a pestle (D). Press-fitted implant into the iliac crest (E). Uneventful wound healing without excessive scar formation after 12 weeks over the left iliac wing (F). Post-operative X-ray showing complete filling of the iliac crest defect after tricortical bone graft harvesting with correct placement of the scaffold biomaterial (G) with clearly visible bone-implant interface (arrow). Post-operative X-ray after 8 months revealing fragmentation and good bony integration of the scaffold with bone bridging of the bone-implant interface region (arrow) from the surrounding host bone into the implant (H).

In all patients, the defects at the iliac crest were filled with blocks of the Greenbone scaffold biomaterial with an average size of 26.3 × 16.8 × 10 mm (length, height, width; range: 15 × 15 × 10 to 40 × 20 × 10 mm). The blocks, which have a standard size of 40x20x10 mm were customized to the individual size of the defect with standard surgical instruments, such as oscillating bone saws and a bone nibbler, were press-fitted into the defect. Care was taken during the press-fit insertion not to fracture the scaffold or the iliac crest region with the gentle use of a pestle and a hammer (see Supplement file). The scaffold could be inserted with good primary stability without fracturing the material itself or the iliac crest (Fig. 1).

All patients showed undisturbed wound healing at the iliac crest without post-operative hematoma or any other biomaterial complication. Post-operative X-rays showed correct positioning of the scaffold in the defect area crest with restoration of the iliac crest after tricortical bone graft harvesting (Fig. 1).

After an average post-operative follow-up of 9.8 months (range: 6–16 months), the visual analog scale (VAS) pain scoring was assessed [13]. Here, n = 7 patients (77.7 %) reported no pain (VAS: 0) and two patients only mild pain (VAS: 1 and VAS: 3). There was no post-operative hematoma, surgical revision or other implant-related complications at the iliac crest. In the second centre, the quality of life of all three recruited patients was assessed using the EQ-5D and revealed a value of 5 (excellent quality of life) for all 3 individuals after 12 months.

**Fig. 2:**
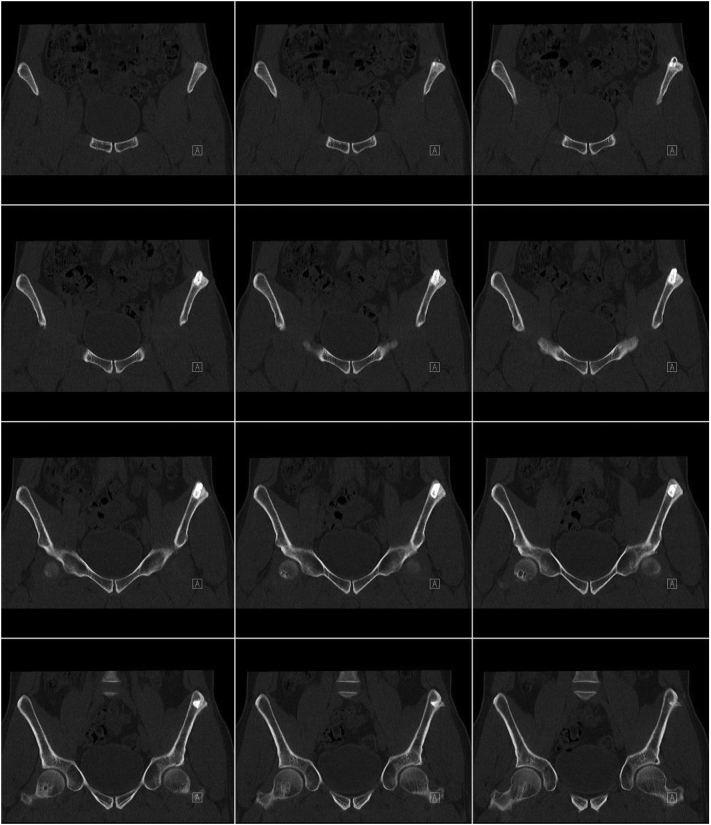
Series of coronal reconstruction CT images of the pelvis 12 months after defect filling at the left iliac crest with a 15 × 15 × 10 mm scaffold block from anterior to posterior. The series shows complete bony integration of the biomaterial into the surrounding host bone with bridging of the bone-implant interface with newly formed bone.

In all patients, good radiographical integration without dislocation of the implant and new bone formation activity with bony integration over time was observed (Fig. 1). Fragmentation of the scaffold as a sign of degradation of the material was also visible. CT imaging revealed bone ingrowth at the bone biomaterial interface as sign of the good osteoconductive properties of the scaffold (Fig. 2).

## Discussion

To the best of our knowledge, this is the first case series reporting the use of a novel rattan-wood based not-sintered hydroxyapatite and beta-tricalcium phosphate material for defect filling after harvesting of tricortical bone graft from the iliac crest.

Although morbidity is an important issue, the iliac crest remains the most frequently harvested donor site for the enhancement of impaired fracture consolidation or reconstruction of bone defects [14]. In the literature, complication rates up to 19.4 % are reported including pain at the donor site as the most common one, followed by infection, hematoma formation, fractures, or soft tissue herniation [14], [15]. Some studies have indicated that a reconstruction of the iliac crest defect significantly decreases such issues, and hence, various bone filling materials have been tested [16], [17]. For instance, defect reconstruction using bone cement and cancellous screws has been reported [18]. Here, the GreenBone biomaterial offers the advantages of absorption and bio-resorption properties with the potential of bone regeneration in the iliac crest defect area [7], [9]. In particular, the GreenBone scaffold biomaterial reproduces the hierarchical architecture and morphology of natural human bone, whereby biomimetic structure drives new bone formation and remodelling [6], [19]. In accordance, good radiographical integration without dislocation of the implant and new bone formation activity was observed in all patients with good bridging of the biomaterial-bone interface with newly formed bone. This might be explainable by the fact that the GreenBone biomaterial is not limited to a particular shape to reconstruct the anatomical site of the iliac crest but can be customized intraoperatively for press-fit implantation of the implant into the defect. This permits restoration of the iliac crest anatomy and provides sufficient primary stability of the biomaterial to allow for further bony integration. Regarding the degradation behavior of the material, the radiographical analysis showed partial fragmentation and slow degradation of the scaffold over time. Fragmentation of the GreenBone implant does most likely enable and facilitate bone ingrowth of the surrounding host bone into the scaffold and slow degradation can be explained by the high amount of hydroxyapatite with only limited degradation potential. Both the fragmentation and the low degradation is in line with findings from a pre-clinical animal trial in a critical-size long bone defects that also revealed partial fragmentation in combination with slow resorption of the material with good bone ingrowth of the surrounding host bone [5].

Further, in all patients, good functional outcomes without acute or late post-operative complications, such as post-operative hematoma, herniation etc., with no or only mild pain were found. Three patients from one centre that evaluated quality of life showed a perfect score of 5 in the EQ-5D questionnaire.

However, some limitations have to be considered. First, this case series included only nine patients without a control group and second, the follow-up time was relatively short, which hinders generalizability of the presented findings. In addition, only patients undergoing tricortical bone graft harvesting from the iliac crest were included. As the iliac crest is not affected by weight-bearing and depicts a well vascularized bone, the clinical use in other less favourable sites or conditions (e.g. diaphyseal bone, established non-union etc.) has to be further investigated.

## Conclusions

The use of the rattan-wood based not-sintered hydroxyapatite and beta-tricalcium phosphate biomaterial for iliac crest defect filling was associated with good clinical outcome, none to mild pain, and radiographic bony incorporation. GreenBone defect filling of iliac crest defects can therefore be regarded as reasonable treatment option to avoid complications after iliac crest harvesting These promising first clinical results should encourage orthopaedic surgeons to further clinically evaluate GreenBone as promising biomaterial for bone defect reconstruction at other anatomical locations.

The following is the supplementary data related to this article.

Video 1 Video file with intraoperative implantation of the scaffold into the iliac crest. Video 1

## Conflict of interest

VA, TB and MP are paid consultants for GREENBONE ORTHO S.p.A., 48018 Faenza (RA), Italy.
